# Integrating vector control across diseases

**DOI:** 10.1186/s12916-015-0491-4

**Published:** 2015-10-01

**Authors:** Nick Golding, Anne L. Wilson, Catherine L. Moyes, Jorge Cano, David M. Pigott, Raman Velayudhan, Simon J. Brooker, David L. Smith, Simon I. Hay, Steve W. Lindsay

**Affiliations:** Wellcome Trust Centre for Human Genetics, University of Oxford, Oxford, OX3 7BN, UK; School of Biological and Biomedical Sciences, Durham University, Durham, DH1 3LE UK; Department of Infectious and Tropical Diseases, London School of Hygiene & Tropical Medicine, London, WC1E 7HT UK; Department of Control of Neglected Tropical Diseases, World Health Organization, 1211 Geneva, Switzerland; Institute of Health Metrics and Evaluation, University of Washington, Seattle, WA 98121 USA; Department of Zoology, University of Oxford, Oxford, OX1 3PS UK; Fogarty International Center, National Institutes of Health, Bethesda, MD 20892 USA

**Keywords:** Disease mapping, Public health, Vector-borne disease, Vector control

## Abstract

**Background:**

Vector-borne diseases cause a significant proportion of the overall burden of disease across the globe, accounting for over 10 % of the burden of infectious diseases. Despite the availability of effective interventions for many of these diseases, a lack of resources prevents their effective control. Many existing vector control interventions are known to be effective against multiple diseases, so combining vector control programmes to simultaneously tackle several diseases could offer more cost-effective and therefore sustainable disease reductions.

**Discussion:**

The highly successful cross-disease integration of vaccine and mass drug administration programmes in low-resource settings acts a precedent for cross-disease vector control. Whilst deliberate implementation of vector control programmes across multiple diseases has yet to be trialled on a large scale, a number of examples of ‘accidental’ cross-disease vector control suggest the potential of such an approach. Combining contemporary high-resolution global maps of the major vector-borne pathogens enables us to quantify overlap in their distributions and to estimate the populations jointly at risk of multiple diseases. Such an analysis shows that over 80 % of the global population live in regions of the world at risk from one vector-borne disease, and more than half the world’s population live in areas where at least two different vector-borne diseases pose a threat to health. Combining information on co-endemicity with an assessment of the overlap of vector control methods effective against these diseases allows us to highlight opportunities for such integration.

**Summary:**

Malaria, leishmaniasis, lymphatic filariasis, and dengue are prime candidates for combined vector control. All four of these diseases overlap considerably in their distributions and there is a growing body of evidence for the effectiveness of insecticide-treated nets, screens, and curtains for controlling all of their vectors. The real-world effectiveness of cross-disease vector control programmes can only be evaluated by large-scale trials, but there is clear evidence of the potential of such an approach to enable greater overall health benefit using the limited funds available.

**Electronic supplementary material:**

The online version of this article (doi:10.1186/s12916-015-0491-4) contains supplementary material, which is available to authorized users.

## Background

### The global impact of vector-borne diseases

Vector-borne diseases impose a significant burden on human health and economic development [1, 2]. Despite the vast scale of the problem, effective interventions are available to control many of these diseases and, therefore, reducing their burden is an achievable public health goal.

**Fig. 1:**
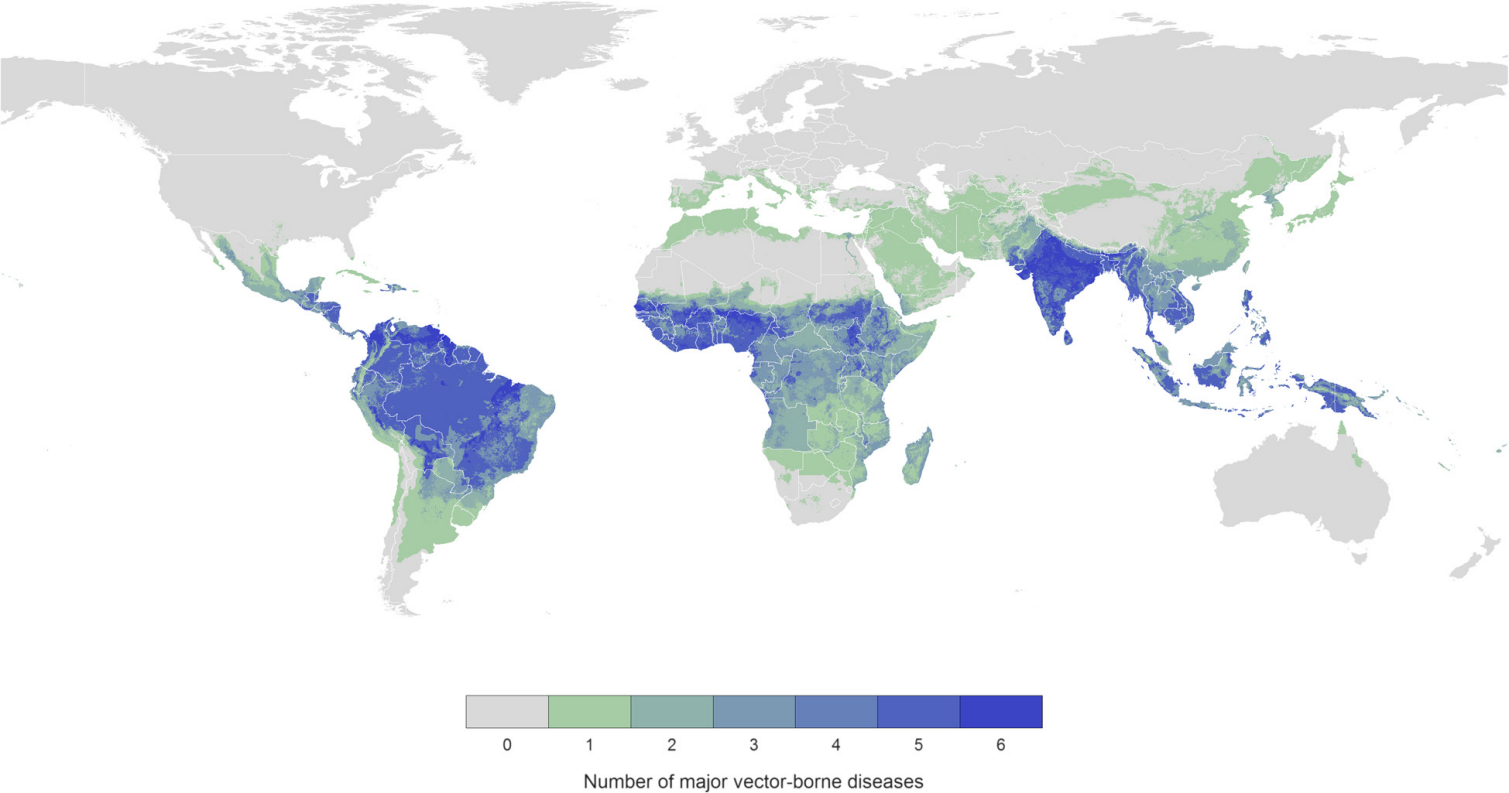
Combined global distribution of seven major vector-borne diseases for which integration of vector control programmes may be beneficial: malaria, lymphatic filariasis, leishmaniasis, dengue, Japanese encephalitis, yellow fever, and Chagas disease. Colours indicate the number of vector-borne diseases that pose a risk at each 5 × 5 km grid cell

Nearly 82 % of the global population live in areas at risk from one vector-borne disease with over half living in areas at risk of two or more of the major vector-borne diseases (see Additional file 1 for further details). The majority of this burden affects those living in low-income countries where resources for disease control are limited. Inhabitants of some parts of sub-Saharan Africa, south Asia, and the Americas are at risk from five or more major vector-borne diseases (Fig. 1). This overlap in the geographic distribution of the major vector-borne diseases suggests it is possible to leverage control resources to tackle these diseases simultaneously [3]. Herein, we identify the populations at risk of multiple major vector-borne diseases to highlight and quantify the opportunity for integrating vector control across these diseases.

### Integrating vector control methods

For many vector-borne diseases, vector control (targeting the arthropods that transmit the disease) is a highly effective means of reducing transmission. For some diseases, such as dengue and Chagas disease, vector control is the only approach currently available [4].

Large-scale vector control programmes have previously been responsible for the near-elimination of river blindness from much of West Africa and for shrinking the range of Chagas disease in South America [5, 6]. Vector control is currently carried out on a massive scale for the control of malaria, with long-lasting insecticidal nets (LLINs) and indoor residual spraying of insecticides reducing the burden of this disease in highly endemic parts of sub-Saharan Africa [7, 8].

Although progress has been made in reducing vector-borne disease endemicity, sustaining and advancing these gains requires intensification of control efforts [9]. Vector-borne disease control is hindered by dwindling financial resources as well as other challenges such as development of insecticide resistance [10]. Simultaneous deployment of multiple vector control methods, some of which are not based on insecticides, can reduce disease transmission to far lower levels than those achieved using a single intervention and help slow the development of insecticide resistance, thereby providing cost-effective and sustainable reductions in disease burden [11]. In areas with multiple vector species, insecticide resistance management programmes should be adopted as soon as resistance is detected in one vector species, although ideally such programmes should be introduced before resistance develops [12].

The simultaneous use of multiple methods is now the preferred vector control strategy and forms a cornerstone of integrated vector management – a best-practice framework for sustainable and cost effective vector control [13].

## Discussion

### Integrating control across diseases

In addition to integrating multiple vector control methods to target a single disease, simultaneously targeting multiple diseases using the same vector control programme infrastructure and possibly the same interventions has the potential for economies of scale and scope and even greater increases in cost-effectiveness. No large-scale vector control programmes have trialled multi-disease vector control, but several examples of ‘accidental’ control of vector-borne diseases suggest the potential of this approach.

Campaigns to reduce the incidence of malaria in India in the 1950s by indoor residual spraying of insecticides are credited with drastically reducing the burden from visceral leishmaniasis by killing its sand fly vectors as they rested inside homes [14]. Similarly, the mass rollout of LLINs in sub-Saharan Africa over the last decade is thought to have reduced the incidence of lymphatic filariasis, since these diseases share the same mosquito vector in rural areas [15].

Combining vector control activities across vector-borne diseases has the potential to be more cost-effective than parallel programmes. Cost savings would come both from reducing the direct costs of deploying interventions and from sharing the necessary support structures for these control programmes.

### Integrated vaccination and mass chemotherapy

This integration of disease control programmes is not without precedent. For example, the initiation in 1974 of the Expanded Programme on Immunization was a ground-breaking move to combine the control programmes for several vaccine-preventable diseases into a single, large-scale programme. By simultaneously deploying vaccines for a range of diseases, and combining the support structures required for large-scale immunization programmes, the Expanded Programme on Immunization was able to slash the costs of controlling each disease [16]. The subsequent development and rollout of polyvalent vaccines has further compounded these savings, enabling even cheaper and more effective disease control. Indeed, these vaccine distribution networks and other public health programmes have already been used as a cost-effective and equitable method for distributing LLINs [17, 18].

The successes of integrated vaccination programmes have recently been mirrored in the integration of mass chemotherapy control programmes for a number of neglected tropical diseases [19]. As with vector control, many of the drugs used in mass chemotherapy are efficacious against multiple diseases. The distribution mechanisms for these drugs are very similar and they can safely be administered together, meaning that cost savings can be made by administering multiple drugs in a single treatment round.

### Identifying opportunities for integration

A crucial first step in assessing where and when an integrated approach to vector-borne disease control is likely to be effective is determining which interventions are effective against which diseases. Robust experimental studies of the effectiveness of vector control methods are unfortunately scarce for most diseases other than malaria [20, 21]. However, the limited studies available suggest that many vector control interventions are effective against several different vector-borne diseases [22].

For example, the most obvious candidates for synergy with malaria vector control methods are lymphatic filariasis (spread by the same mosquito vectors as malaria in rural Africa) and leishmaniasis (spread by the bite of sand flies). There is good evidence for the efficacy of insecticide-treated nets, screens, and curtains against both of these diseases, as several of the main vector species share the house-entering behaviour of the most important malaria vectors [23, 24]. There is also limited evidence for the effectiveness of these methods at controlling the mosquito vectors of dengue and yellow fever, and of Japanese encephalitis [25–28]. Indoor residual spraying of insecticides is likely to be effective against leishmaniasis and lymphatic filariasis vectors [29, 30] and is known to be highly effective at controlling the Triatomine bug vectors of Chagas disease [31].

Larval source management is an effective (if not widely used) tool for the control of malaria [32] and can be combined with other vector control approaches for an additive reduction in malaria burden [33]. Similarly, larval control has been successfully combined with mass drug administration for the control of lymphatic filariasis in India [34]. Control of larval *Aedes* mosquitoes is a widely used intervention for tackling dengue [35] and was historically a key tool in the elimination of both dengue and yellow fever from Cuba and Panama [36].

Whilst insecticide treatment of nets, screens, and walls are implemented at the household level, larval source management must be targeted at the breeding sites of the specific vector species of interest, necessitating different procedures for different vectors. For example, application of larvicides is appropriate for controlling *Anopheles* [33], whereas polystyrene beads may be more effective for the urban *Culex* vectors of lymphatic filariasis [34] and the removal or larvicidal treatment of water containers is more useful for the *Aedes* vectors of dengue and yellow fever [35, 37]. Nevertheless, there are many situations where *Anopheles* and *Culex* mosquitoes share the same habitat [38, 39], and control operatives could reasonably be tasked to treat or remove the distinct larval habitats of several key species in a single programme, sharing many of the costs of control. The development of larval control products that are effective against multiple vectors could enhance these cost savings. Evaluating the practical feasibility and quantifying the cost-effectiveness of such cross-disease integration of larval source management should be considered in detail in future studies.

For many vector-borne diseases, improving the quality of housing can be an effective method of disease control [40, 41]. Whilst house improvement can mean different things in different epidemiological situations (such as screening roof spaces for malaria control [42, 43] versus repairing plaster for Chagas disease [44, 45]), integrated programmes that carry out multiple improvements could be an effective approach for jointly controlling multiple diseases.

The likely impacts of applying control methods simultaneously against multiple vectors are a cause of debate amongst vector ecologists. However, a deliberate integration programme has so far never been applied operationally or evaluated in a research context. Therefore, evaluation of programmes targeting multiple vectors and diseases should be a priority for future research in order to determine the effectiveness, cost-effectiveness, and feasibility of this approach.

## Summary

### Quantifying the opportunity for integration of vector control

**Fig. 2:**
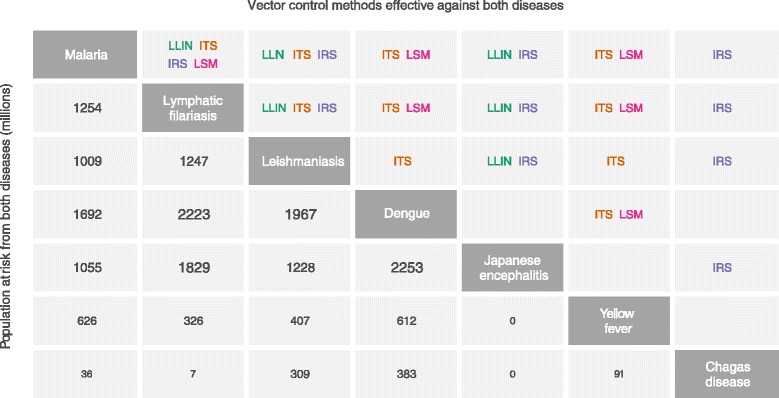
Effective vector control interventions and joint population at risk of pairs of vector-borne diseases. To assess the potential for integrating vector control between a pair of diseases, the two diseases in the diagonal cells are identified, followed by the cells where their rows and columns intersect. Cells in the lower left give the number of people (in millions) living in areas at risk from a given pair of diseases. Cells in the upper-right list vector control methods which may be effective against both diseases (see Additional file 1 for details). LLINs, Long-lasting insecticidal nets; ITS, Insecticidal house screening or curtains; IRS, Indoor residual spraying of insecticides; LSM, Larval source management. Whilst some of these vector control methods can be deployed in exactly the same way for multiple diseases (e.g. LLIN for malaria and lymphatic filariasis) and can therefore be easily targeted at multiple diseases, others will require different procedures for different diseases (e.g. LSM for malaria and dengue) and the potential for combined control will be more limited

Quantifying the potential for cross-disease integration of vector control requires assessment of the overlap between vector-borne diseases, both in terms of populations affected and in the potential for integration of control. Using contemporary high-resolution risk maps we estimated the populations at risk from pairs of major vector-borne diseases. Figure 2 compares these figures with vector control methods that are likely to be effective against both diseases. Malaria, leishmaniasis, lymphatic filariasis, and dengue are prime candidates for joint control due to their significant public health impact, broad global distribution (populations at risk per pair of these diseases range between 1 and 2 billion people), and susceptibility to proven vector control methods. Of the 3.9 billion people living in areas at risk from at least two of the seven vector-borne diseases considered here, 3.5 billion (90 %) inhabit regions where two or more of these diseases are likely to be susceptible to the same intervention.

These global maps indicate where diseases are likely to overlap in their distributions, but diseases may not overlap at finer scales if their vectors have distinct environmental requirements. Large-scale cross-disease vector control programmes would need to be adapted to local-scale variation in order to best target the specific combination of diseases present in at-risk communities. Planning a large-scale programme of integrated vector control will therefore require more detailed knowledge of the spatial distribution of each disease, as well as their susceptibility to available vector control methods. As with any vector control programme, the interventions selected for control would need to be tailored to the local environment as well as to the vector species present. The operational effectiveness of multi-disease vector control programmes must then be evaluated in the field.

Whilst these hurdles mean that cross-disease integration of vector control may not be feasible in all of these areas, the scale of the potential public health gains is sufficiently large to warrant serious attention and future research. Given the limited funds available to control vector-borne diseases [10], successfully reducing the burden of these diseases requires new strategies to implement vector control cost-effectively. Leveraging vector control programmes in a single framework to attack multiple diseases promises a greater overall health benefit using the funds available. Mapping the global distribution of the populations suitable for cross-disease integration of vector control is the first step in quantifying the potential public health dividend.

## Additional file

Additional file 1:
**Details of: selection of major vector-borne diseases and vector control interventions for evaluation, compilation of risk maps for these diseases, and calculation of joint population at risk estimates.** (DOCX 2809 kb)

## Abbreviation

LLINs: Long-lasting insecticidal nets
