# Supplementary material for: Integrating vector control across diseases
Source: BMC Med. 2015 Oct 1;13:249. doi: 10.1186/s12916-015-0491-4 (PMC4590270; doi:10.1186/s12916-015-0491-4)
Supplement: Additional file 1: — Details of: selection of major vector-borne diseases and vector control interventions for evaluation, compilation of risk maps for these diseases, and calculation of joint population at risk estimates. (DOCX 2809 kb) [file 12916_2015_491_MOESM1_ESM.docx]

# Supplementary Materials:

This file contains supporting information for the manuscript *Integrating vector control across diseases* by Golding *et al.* and details selection of major vector-borne diseases and vector control interventions for evaluation, compilation of risk maps for these diseases and calculation of joint population at risk estimates.

## *Selection of major vector-borne diseases for evaluation*

Vector-borne diseases encompass a range of diseases with varying epidemiologies and magnitudes of disease burden. Since our aim was to produce a first quantification of the potential for cross-disease integration of vector control, we focussed only on those which cause the largest disease burden, for which sufficient data were available to map disease risk and for which cross-disease integration of vector-control would be plausible.

The World Health Organization’s Handbook for Integrated Vector Management (*1*) considers 10 specific vector-borne diseases: malaria, lymphatic filariasis, dengue, schistosomiasis, leishmaniasis, Chagas disease, trachoma, onchocerciasis, Japanese encephalitis and human African trypanosomiasis. Of these, we excluded four diseases: schistosomiasis, trachoma, onchocerciasis and African trypanosomiasis. All of these diseases are transmitted by species with distinct behaviour and ecology (respectively fresh water snails; the human face fly *Musca sorbens*; blackflies and tsetse flies) meaning that there is no substantial overlap in vector control methods with other major vector-borne diseases. We additionally include yellow fever since it shares its principal vector, *Aedes aegypti* with dengue and is therefore an important candidates for cross-disease integration of vector control. We did not include Chikungunya (which also shares vector species with dengue) as a contemporary map of the global distribution of this disease was not available.

These 7 major vector-borne diseases have a significant public-health impact, with a combined burden of over 90 million disability-adjusted life years. This accounts for approximately 83% of the total global burden of malaria and the neglected tropical diseases: 108.7 million disability-adjusted life years (2).

## *Risk maps of the major vector-borne diseases*

### *Malaria*

We consider here the two major forms of malaria, caused by the parasites *Plasmodium falciparum* and *P. vivax*. Whilst three other parasites (*P. ovale*, *P. malariae* and *P. knowlesi*) also cause human malaria, they largely exist within the extents of the two major parasites and detailed information on their distributions is currently unavailable.

Contemporary global maps of the prevalence of *P. falciparum* and *P. vivax* malaria have been published by (*3*) and (*4*). In both of these studies the first stage of map production was defining the spatial limits of transmission risk. These limits were defined as areas where transmission was stable (annual parasite incidence of at least 0.1 per 1,000 *per annum*), unstable (less than 0.1 per 1,000 *per annum*) or no risk. This classification was based on national reporting of malaria cases as well as temperature masks to exclude areas where malaria transmission would not be possible, and an aridity mask downgrading the class of risk.

We used a presence/absence map of malaria occurrence (combining *P. falciparum* and *P. vivax*) to enable comparison with distribution maps of other vector-borne diseases for which prevalence information was unavailable. Risk was defined as areas of stable transmission (annual parasite incidence of at least 0.1 per 1,000 *per annum*) of either parasite and all other areas were classed as not at risk. The choice of threshold was motivated by an assumption that vector control interventions would most likely be used across areas with stable risk whereas resources in unstable areas are targeted at imported cases and the containment of local outbreaks (*5*). The resulting risk map for malaria is given in Figure S1.


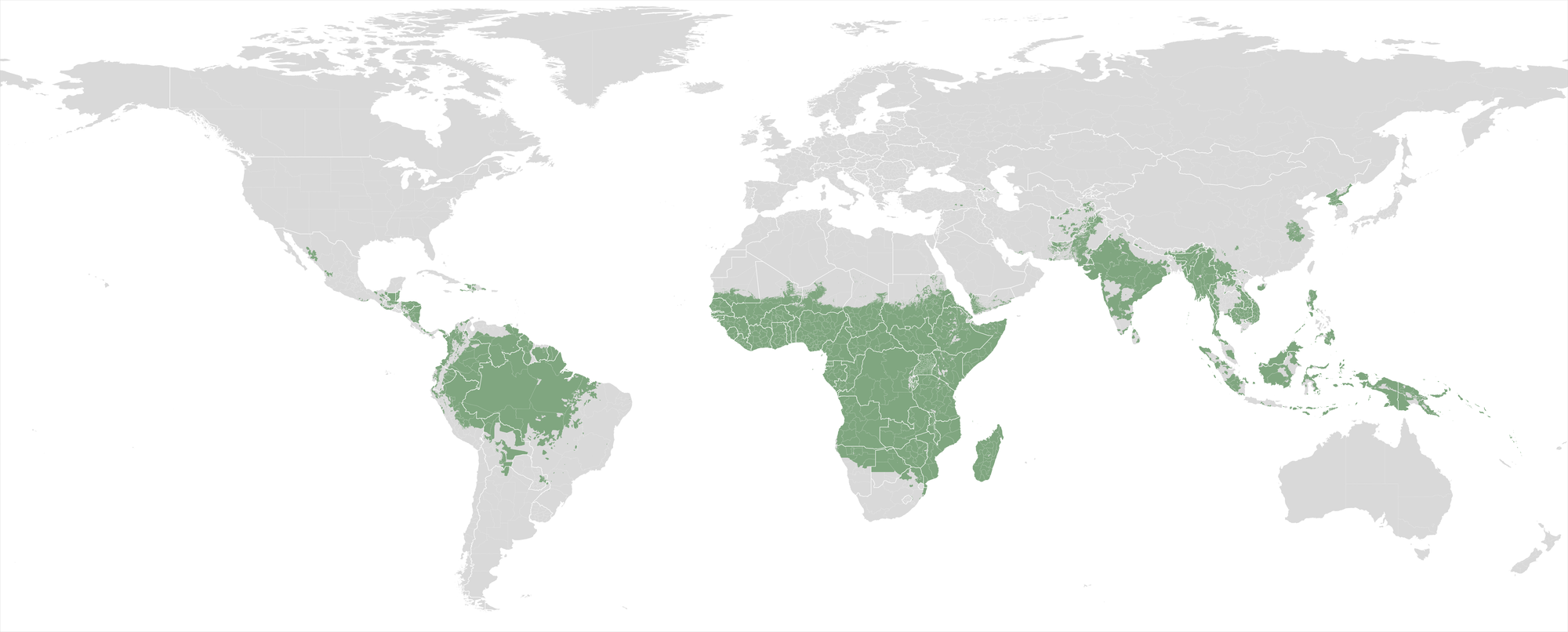


Figure S1. The global distribution of malaria (Plasmodium falciparum or P. vivax) transmission risk. The green area shows the limits of stable transmission.

### *Dengue*

Mapping the risk of this disease required collation of records of occurrence of the disease from multiple sources and mapping using an environmental modelling approach based on species distribution modelling approaches widely used in ecology (*6*). This analysis and the resulting map of environmental risk of the disease are described in detail in (*7*) but we outline the process briefly here.

Three components were required to construct the disease distribution model: a global database of 8,309 spatially and temporally unique records of the occurrence of dengue; a map of the consensus of evidence for and against the presence of dengue for all countries of the world (*8*); a dataset of six global, gridded environmental covariates: precipitation; suitability of temperature for dengue transmission; remotely-sensed normalized vegetation index; relative poverty; travel time to a major city; and urbanisation. The evidence consensus layer was used to generate ‘pseudo-absence’ locations (where the disease had not been reported, biased towards countries where the disease is most likely to be absent) and these were used to train an ensemble of boosted regression trees (BRT) statistical models (9). This BRT ensemble model and the global environmental covariate layers were then used to predict environmental risk of dengue within those countries where the consensus of evidence was that dengue was or could be present. The resulting risk map displayed a continuous metric of the risk of dengue transmission.

In order to convert this continuous map into a binary risk map, it was necessary to select a threshold level of the risk metric above which to classify areas as at-risk. To do this, the predicted risk metric was recorded for the locations of each known occurrence record and the risk threshold set at the value of environmental risk above which 95% of occurrence records were classified as at-risk. We used a threshold of 95% (rather than 100%) to account for the potential errors in the occurrence database which could have occurred due to incorrect geopositioning of records or undetected importation of cases from environmentally suitable areas. Comparing this and other quantiles of environmental risk with the evidence consensus layer supported our choice of this 95% level. The resulting global presence/absence dengue distribution map is shown in Figure S2.


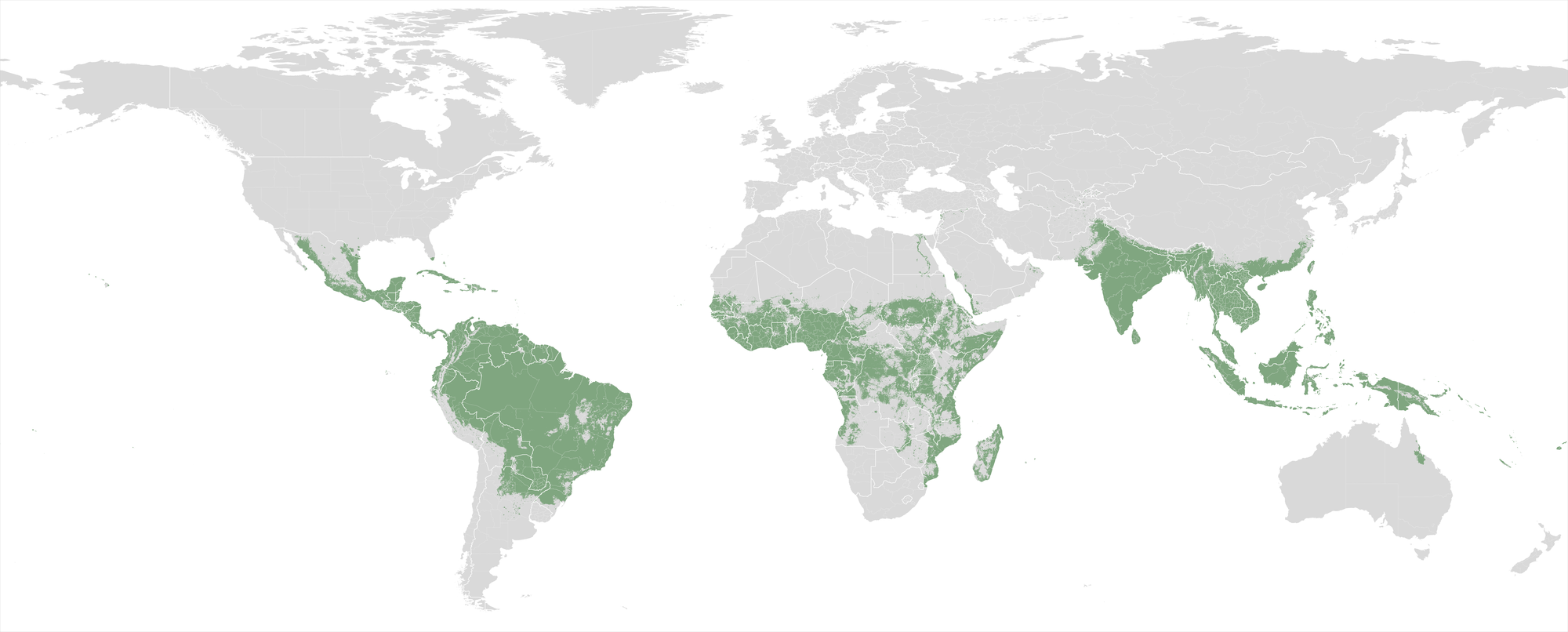
Figure S2. The global distribution of dengue. The green area shows the limits of risk, defined by applying a threshold to the map of (*7*).

### *Leishmaniasis*

The global distribution of leishmaniasis was mapped on a global scale using a similar approach to that used for dengue and is described in more detail in (*10*) though we provide a brief summary here.

Constructing global maps for leishmaniasis is complicated by the existence of multiple species of *Leishmania* parasite, multiple vector species and multiple clinical forms, with stark epidemiological differences between the Old World and New World forms of the disease. To account for these differences separate maps were produced for the cutaneous (including mucosal leishmaniasis; a total of 6,426 occurrence records) and visceral (including post-kala-azar dermal leishmaniasis; 6,137 occurrence records) forms and for each of these maps, separate models fitted for the New and Old Worlds, resulting in four separate distribution models, each fitted using the BRT modelling approach used for modelling dengue. Whilst each of these four subgroups of leishmaniasis eco-epidemiology represents multiple combinations of vectors, parasites and clinical presentations, the flexible statistical framework used is able to model complex functions of environmental covariates and can therefore capture the effects of multiple different diseases cycles in a single model (*9*). The resulting global environmental risk maps for cutaneous and visceral leishmaniasis were converted into binary maps of at-risk and not-at-risk regions for each disease following the same procedure as for dengue. For both diseases the threshold was set so that 90% of occurrence records fell in at-risk areas. This threshold again compared favourably to the evidence consensus layers produced for each disease as well as national-level estimates of disease incidence (*11*). The two maps were combined to create a single map of the distribution of leishmaniasis (areas where risk is posed by at least one form of the disease), shown in Figure S3.


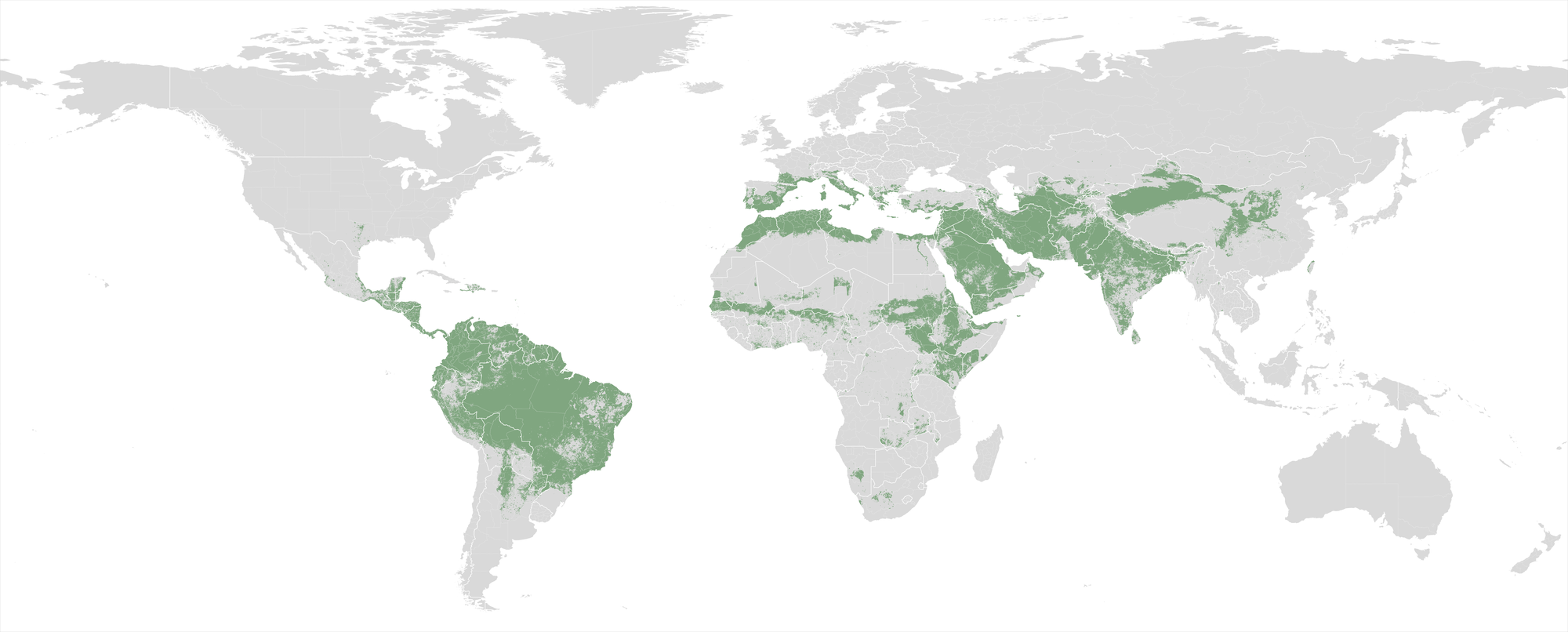
Figure S3. The global distribution of leishmaniasis. The green area shows the limits of risk, defined by (*9*) using an environmental modelling approach.

### *Lymphatic filariasis*

The global distribution of lymphatic filariasis was mapped on a global scale using a similar approach to that used for dengue and leishmaniasis and is described in more detail in (*12*). Again, a brief summary follows.

An initial national-level global distribution map of the disease was constructed, based on data assembled in the Global Atlas of Helminth Infection ([www.thiswormyworld.org](http://www.thiswormyworld.org/)) to identify countries in which transmission is either known or suspected. This national-level map was then refined using statistical modelling. A comprehensive database was assembled of lymphatic filariasis 8,106 prevalence surveys conducted between 1902 and 2013 in 86 countries historically or currently known to be endemic for the disease. These prevalence data were converted into presence records (in which at least one case was recorded) and absence records (in which no cases were recorded). An ensemble BRT modelling approach was then used to map environmental suitability for lymphatic filariasis transmission based on environmental variables known to be related to development of both the parasites and their vectors.

In order to convert this map of environmental suitability into a presence/absence outlining the limits of transmission, a threshold of suitability was determined, above which transmission was be assumed to be possible. Unlike for dengue and leishmaniasis, a sufficient number of geolocated records of the absence of transmission were available to empirically determine an optimal suitability threshold. The threshold was selected to maximise three goodness-of-fit metrics of the resulting binary classification against the original disease occurrence dataset. These metrics were: sensitivity; specificity and the proportion of records from the occurrence dataset which were correctly classified as either presence or absence by the presence/absence map.

Finally, the resulting occurrence map was masked on a map of current endemicity in order to reflect contemporary limits of transmission (Figure S4).


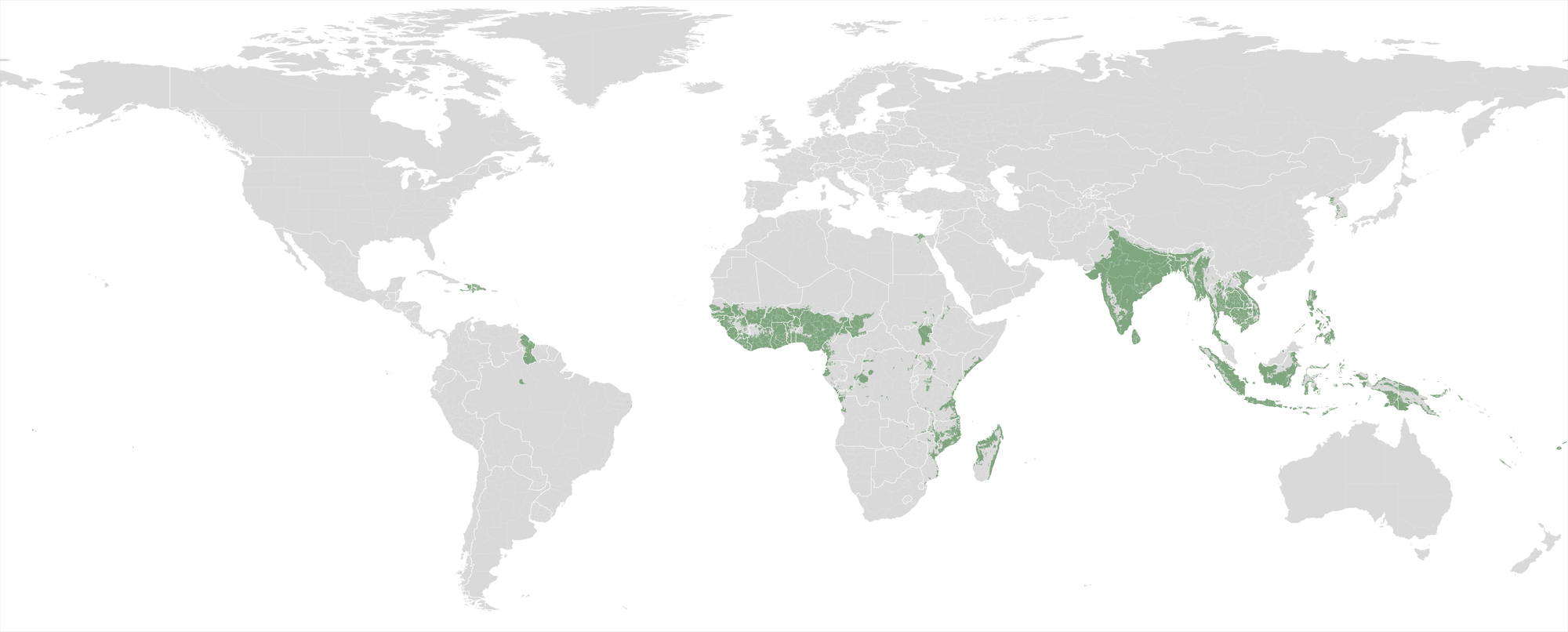
Figure S4. The global distribution of lymphatic filariasis. The green area shows the limits of risk, as defined by (*12*) using an environmental modelling approach.

### *Yellow fever*

Yellow fever is broadly distributed across Central and South America and sub-Saharan Africa. A map of the current extent of the disease, at a sub-national level was obtained from the US Centers for Disease Control (*13*). This map was manually georeferenced and digitised and is presented in Figure S5. This map largely concurs with the distribution of the disease in Africa predicted by (*14*) - also at a sub-national level. At present, insufficient information is available to increase the spatial precision of this map.


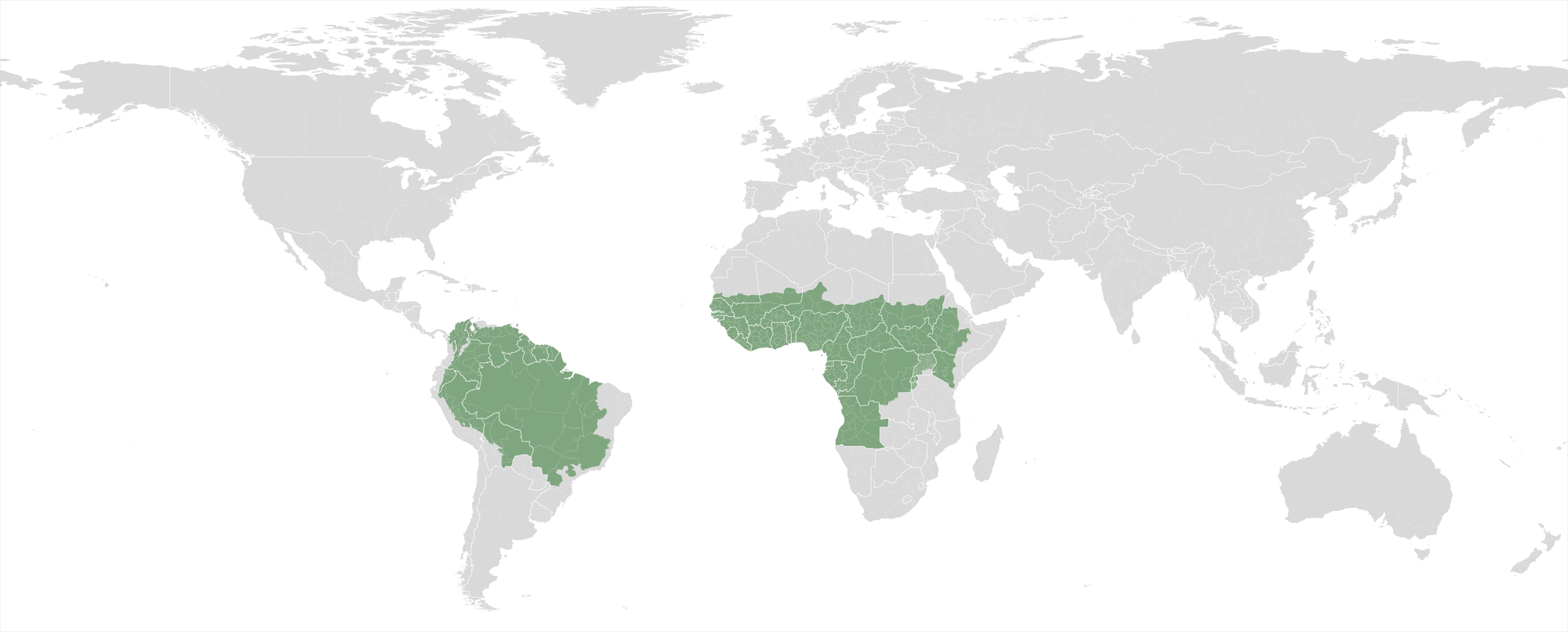
Figure S5. The global distribution of yellow fever. The green area shows the current geographic extent of the disease, digitised from (*13*).

### *Japanese encephalitis*

Japanese encephalitis is restricted in its distribution predominantly to south and south-east Asia and the Far East. A map of the geographic extent of all known cases was obtained from the US Centers for Disease Control (*15*), manually georeferenced and digitised. This extent map includes large areas of varying habitat type, many of which are unlikely to be at risk of Japanese encephalitis due to the absence of the major mosquito vector *Culex tritaeniorhynchus*. This extent layer was refined using a published map of the predicted distribution of *Cx. tritaeniorhynchus*, previously used as a proxy for Japanese encephalitis risk (*16*). Since this predictive vector map was available as a continuously index of risk, a procedure similar to that applied for dengue and leishmaniasis was used to convert it into a binary map of the predicted presence or absence of the vector. Using a dataset of 7,701 records of the occurrence of Japanese encephalitis collated from published literature and online reports, a threshold risk value was defined which categorised 95% of these occurrences as falling in areas where the vector is predicted to be present. The resulting binary map of risk of Japanese encephalitis shows areas where the vector is predicted to be present, which are inside the known geographic extent of the disease (Figure S6).


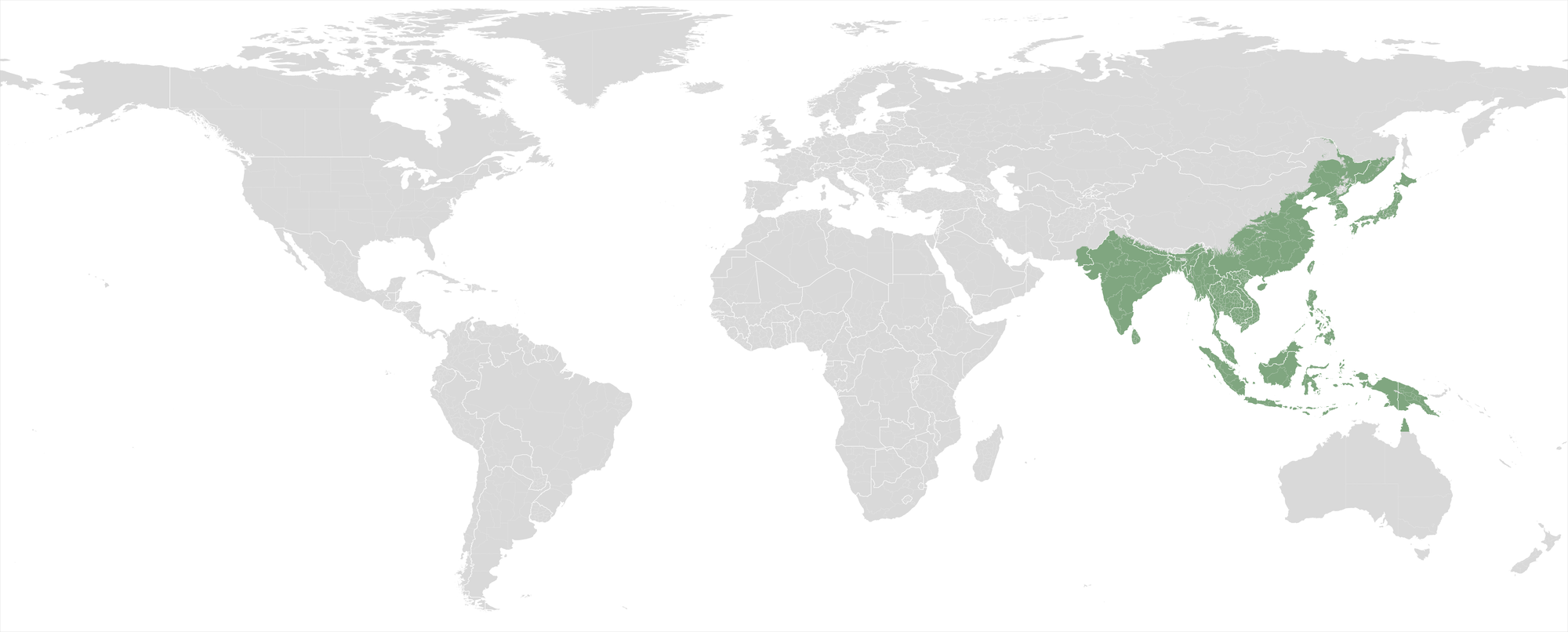
Figure S6. The global distribution of Japanese encephalitis. The green area shows the geographic extent of known occurrences of the disease, digitised from (*15*) and further refined to areas where the principal vector mosquito *Culex tritaeniorhynchus* is likely to be present (*16*).

### *Chagas disease*

Very little information is available on the current global distribution of Chagas disease. Whilst recent national-level maps of the distribution of the disease (*17*) and global distribution maps of some of its vectors (*18*) are available, the most spatially refined and up-to date global information on the global distribution of Chagas disease that could be found was an expert-opinion disease extent map in a 1989 WHO manual (*19*). This map was manually georeferenced and digitised and is presented in Figure S7.


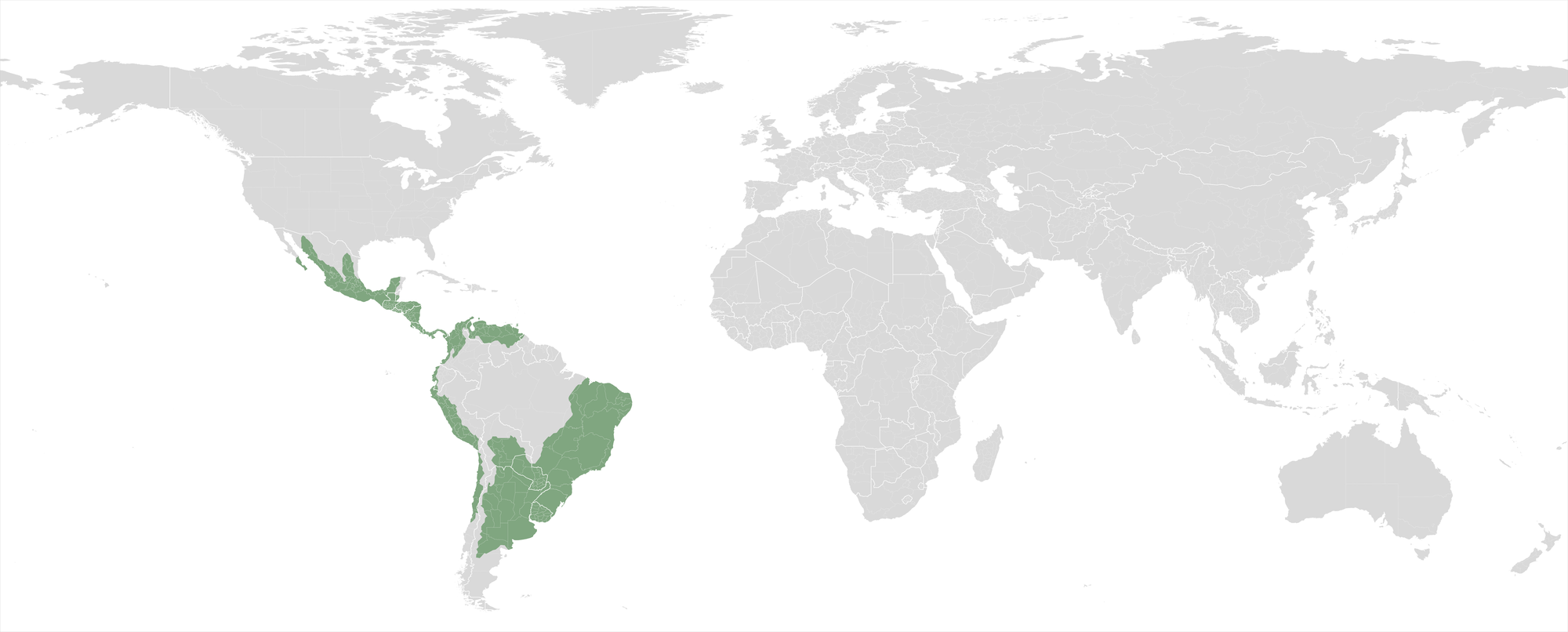
Figure S7. The global distribution of Chagas disease. The green area shows areas at risk of Chagas disease as defined by (19), the most recent published risk map available.

## *Calculation of populations at risk*

All of the risk maps were formatted to the same layout with a spatial resolution (grid cell size) of 5x5km. To estimate the population at risk of each pair of the vector-borne diseases, the grid cells in which both diseases were assessed as posing a risk were identified, and the estimated human population in these cells extracted from the 2010 Gridded Population of the World dataset (*20*, *21*) and summed.

The proportion of the global population at risk from two or more of these 7 major vector-borne diseases considered here was estimated by calculating the population in cells at risk from any pair of these vector borne diseases (3.916 billion) and dividing this by the total estimated global population from the Gridded Population of the World dataset (6.936 billion). This resulted in an estimate of 56.5% of the global population at risk from two or more of these 7 vector-borne diseases.

Similarly, we calculated the population living in areas where at least two of these diseases are present for which a shared intervention (any intervention in Figure 2 in the main text) for these two diseases is available. This resulted in a total of 3.545 billion, 91% of the population at risk of two or more diseases, and 51% of the total global population.

## *Shared interventions across vector borne diseases*

We assessed the strength of evidence for the effectiveness of 4 key vector control methods against each of the 7 vector-borne diseases mapped: long-lasting insecticidal nets (LLIN); insecticidal house-screening or curtains (ITS); indoor residual spraying of insecticides (IRS) and larval source management (LSM).

Published research providing evidence for effectiveness of each intervention against each disease (in at least one setting) was collated from existing systematic reviews, literature known to the authors and from a systematic review of the efficacy of all types of vector control tools against the 7 VBD and/or their vectors (databases and search terms available on request). Our aim was to highlight groups of diseases for which combined vector control should be investigated in more detail, rather than to provide a definitive list of appropriate interventions. Table S1 presents our estimate of the strength of evidence for intervention effectiveness against each disease, along with references to the published research used as evidence.

Table S1: Strength of evidence for effectiveness of vector control interventions against each of the major vector-borne diseases. Plus signs indicate the subjective strength of evidence, from 1 (low) to 3 (high) based on the available literature. References are given in parenthesis below these marks. Intervention-disease combinations for which very little or no published evidence was found are marked with a dash.

|  | Malaria | Lymphatic filariasis | Leishmaniasis | Dengue | Japanese encephalitis | Yellow fever | Chagas disease |
| --- | --- | --- | --- | --- | --- | --- | --- |
| Long-lasting insecticidal nets (**LLIN**) | **+++**  (*22*) | **++**  (*23-26*) | **++**  (*23,27*) | **-** | **+**  (*23*) | **-** | **-** |
| Insecticidal house screening or curtains (**ITS**) | **++**  (*22,28,29*) | **++**  (*28,29*)* | **+**  (*23*) | **++**  (*23*) | **-** | **+**  (*30*) | **-** |
| Indoor residual spraying of insecticides (**IRS**) | **+++**  (*31*) | **++**  (*32-34*) | **++**  (*35-42*) | **-** | **+**  (*43*) | **-** | **+++**  (*44-47*) |
| Larval source management (**LSM**) | **++**  (*48*) | **++**  (*49-53*) | **-** | **++**  (*54*) | **-** | **+**  (*30*) | **-** |

* based on efficacy of this intervention against malaria transmitted by *Anopheles gambiae*, which is also the vector for rural LF in sub-Saharan Africa.

## References:

1. World Health Organization (2012) *Handbook for integrated vector management* World Health Organization, Geneva

2. C. J. L. Murray *et al.* (2012) Disability-adjusted life years (DALYs) for 291 diseases and injuries in 21 regions, 1990-2010: a systematic analysis for the Global Burden of Disease Study 2010, *Lancet* **380**, 2197–223

3. P. W. Gething *et al.* (2011) A new world malaria map: *Plasmodium falciparum* endemicity in 2010, *Malar. J.* **10**, 378

4. P. W. Gething *et al.* (2012) A long neglected world malaria map: *Plasmodium vivax endemicity* in 2010, *PLoS Negl. Trop. Dis.* **6**, e1814

5. World Health Organization (2007) *Malaria elimination: a field manual for low and moderate endemic countries* World Health Organization, Geneva

6. J. Elith & J. R. Leathwick (2009) Species distribution models: ecological explanation and prediction across space and time, *Annu. Rev. Ecol. Evol. Syst.* **40**, 677–697

7. S. Bhatt *et al.* (2013) The global distribution and burden of dengue, *Nature* **496**, 504–507

8. O. J. Brady *et al.* (2012) Refining the global spatial limits of dengue virus transmission by evidence-based consensus, *PLoS Negl. Trop. Dis.* **6**, e1760

9. J. Elith, J. R. Leathwick & T. Hastie (2008) A working guide to boosted regression trees, *J. Anim. Ecol.* **77**, 802–13

10. D.M. Pigott *et al.* (2014). Global distribution maps of the Leishmaniases. *eLife* **3**, e02851

11. J. Alvar *et al.* (2012) Leishmaniasis worldwide and global estimates of its incidence, *PLoS One* 7, e35671

12. J. Cano *et al.* (2014). The global distribution and transmission limits of lymphatic filariasis: past and present, *Parasit. Vectors* **7**, 466

13. Centers for Disease Control (2007) *Yellow fever maps* (available at <http://www.cdc.gov/yellowfever/maps/l>)

14. T. Garske *et al.* (2014) Yellow Fever in Africa: estimating the burden of disease and impact of mass vaccination from outbreak and serological data, *PLoS Med.* **11**, e1001638

15. Centers for Disease Control (2012) *Geographic distribution of Japanese encephalitis virus* (available at <http://www.cdc.gov/japaneseencephalitis/maps/index.html>)

16. R. H. Miller *et al.* (2012) Ecological niche modeling to estimate the distribution of Japanese encephalitis virus in Asia, *PLoS Negl. Trop. Dis.* **6**, e1678

17. P. Mischler *et al.* (2012) Environmental and socio-economic risk modelling for Chagas disease in Bolivia, *Geospat. Health* **6**, S59–66

18. C. Schofield, J. Jannin & R. Salvatella (2006) The future of Chagas disease control, *Trends Parasitol.* **22**, 583-8

19. WHO Division of Vector Biology and Control (1989), *Geographical distribution of arthropod-borne diseases and their principal vectors* World Health Organization, Geneva (available at <http://apps.who.int/iris/handle/10665/60575>)

20. CIESIN/CIAT (2004) *Gridded population of the world (GPW), version 3* (available at <http://sedac.ciesin.columbia.edu/gpw>)

21. D. L. Balk *et al.* (2006) Determining global population distribution: methods, applications and data, *Adv. Parasitol.* **62**, 119–15

22. C. Lengeler (2004) Insecticide-treated bed nets and curtains for preventing malaria *Cochrane Database Syst. Rev.*: CD000363

23. A.L. Wilson *et al.* (2014) Benefit of insecticide-treated nets, curtains and screening on vector borne diseases, excluding malaria: a systematic review and meta-analysis *PLoS Negl. Trop. Dis.* **8**, e3228

24. L.J. Reimer *et al.* (2013) Insecticidal bed nets and filariasis transmission in Papua New Guinea *New Engl. J. Med.* **369**, 745-753.

25. D. Prybylski, W.A. Alto, S. Mengea & S. Odaibaiyue (1994) Introduction of an integrated community-based bancroftian filariasis control program into the Mt Bosavi region of the Southern Highlands of Papua New Guinea *Papua New Guinea Med.* **37,** 82-89

26. A. Eigege *et al.* (2013) Long-lasting insecticidal nets are synergistic with mass drug administration for interruption of lymphatic filariasis transmission in Nigeria. *PLoS Negl. Trop. Dis.* **7**, e2508

27. K. Ritmeijer *et al.* (2007) Evaluation of a mass distribution programme for fine-mesh impregnated bednets against visceral leishmaniasis in eastern Sudan. *Trop. Med. Int. Health* **12**, 404-414

28. M.J. Kirby *et al.* (2009) Effect of two different house screening interventions on exposure to malaria vectors and on anaemia in children in The Gambia: a randomised controlled trial. *Lancet* **374**, 998-1009

29. F.M. Gouissi *et al.* (2012) Contribution of poses screen preimpregnated (PSP) installed at openings and eaves of dwellings in the reduction of malaria transmission in the commune of Aguégués in bénin. *Asian Pac. J. Trop. Med.* **6**, 412-420

30. J.A. Le Prince & A.J. Orenstein (1916*) Mosquito control in Panama; the eradication of malaria and yellow fever in Cuba and Panama*. New York / London, Putnam

31. B. Pluess, F.C. Tanser, C. Lengeler & B.L. Sharp (2010) Indoor residual spraying for preventing malaria. *Cochrane Database Syst. Rev.*: CD006657

32. M.S. Chang, B.C. Ho & K.L. Chan (1991) Efficacy of diethylcarbamazine and pirimiphos-methyl residual spraying in controlling brugian filariasis. *Trop. Med. Parasitol.* **42**, 95-102

33. R.H. Webber (1979) Eradication of *Wuchereria bancrofti* infection through vector control. *Trans. Roy. Soc. Trop. Med. Hyg.* **73**, 722-724

34. M.O.T. Iyengar, H. De Rook & W.J.O.M. Van Dijk (1959) Interruption of transmission of *Anopheles*-borne filariasis by indoor residual spraying in Netherlands New Guinea. *Trop. Geogr. Med.* **11**, 287-290

35. C.R. Davies *et al.* (2000) Spraying houses in the Peruvian Andes with lambda-cyhalothrin protects residents against cutaneous leishmaniasis. *Trans. Roy. Soc. Trop. Med. Hyg.* **94**, 631-636

36. H. Reyburn, R. Ashford, M. Mohsen, S. Hewitt & M. Rowland (2000) A randomized controlled trial of insecticide-treated bednets and chaddars or top sheets, and residual spraying of interior rooms for the prevention of cutaneous leishmaniasis in Kabul, Afghanistan. *Trans. Roy. Soc. Trop. Med. Hyg.* **94**, 361-366

37. M.A. Seyedi-Rashti & A. Nadim (1974) Re-establishment of cutaneous leishmaniasis after cessation of anti-malaria spraying. *Trop. Geogr. Med.* **27**, 79-82

38. A. Corradetti (1954) The fight against leishmaniasis by means of the *Phlebotomus* control in Italy. *Rend.– Ist. Sup. Sanit.* **17**, 374-384

39. A.A. Abbas *et al.* (2013) First determination of impact and outcome indicators following indoor residual spraying (IRS) with deltamethrin in a new focus of anthroponotic cutaneous leishmaniasis (ACL) in Iran. *Asian Pac. J. Trop. Dis.* **3**, 5-9

40. C.R. Davies CR *et al.* (1994) The fall and rise of Andean cutaneous leishmaniasis: transient impact of the DDT campaign in Peru. *Trans. Roy. Soc. Trop. Med. Hyg.* **88**, 389-393

41. A.B. Joshi, L.R. Bhatt, S. Regmi & R.W. Ashford (2003) An assessment of the Effectiveness of Insecticide Spray in the Control of Visceral leishmaniasis in Nepal. *J. Nepal Health Res. Counc.* **1**, 1-6

42. K. Kishore *et al.* (2006) Vector control in leishmaniasis. *Indian J. Med. Res.* **123**, 467-472

43. P.K. Srivastava, A.V. Sadanand, N.B.L. Saxena, R.K. Dasgupta, M.V.V.L. Narasimham (1994) Impact of residual insecticidal spray on the incidence of Japanese encephalitis. *J. Commun. Dis.* **26**, 59-61

44. R. Chuit *et al.* (1989) Usefulness of serology for the evaluation of *Trypanosoma cruzi* transmission in endemic areas of Chagas' disease. *Rev. Soc. Bras. Med. Trop.* **22**, 119-124

45. J.C.P. Dias, A.C. Silveira & C.J. Schofield (2002) The impact of Chagas disease control in Latin America - a review. *Mem. I. Oswaldo Cruz* **97**, 603-612

46. K. Hashimoto & C.J. Schofield (2012) Elimination of *Rhodnius prolixus* in Central America. *Parasit. Vectors* **5**, 45

47. C.J. Schofield & J.C. Dias (1999) The Southern Cone Initiative against Chagas disease. *Adv. Parasitol.* **42**, 1-27

48. L. Tusting *et al.* (2013) Mosquito larval source management for controlling malaria *Cochrane Database Syst. Rev.,* CD008923

49. C.A. Maxwell, K. Mohammed, U. Kisumku & C.F. Curtis (1999) Can vector control play a useful supplementary role against bancroftian filariasis? *Bull. World Health Organ.* **77**, 138-143

50. C.A. Maxwell *et al.* (1990) Control of Bancroftian filariasis by integrating therapy with vector control using polystyrene beads in wet pit latrines *Trans. Roy. Soc. Trop. Med. Hyg.* **84**, 709-714

51. S. Subramanian, S.P. Pani, P.K. Das & P.K. Rajagopalan (1989) Bancroftian filariasis in Pondicherry, south India: 2. Epidemiological evaluation of the effect of vector control *Epidemiol. Infect.* **103**, 693-702

52. R. Reuben *et al.* (2001) Annual single-dose diethylcarbamazine plus ivermectin for control of bancroftian filariasis: Comparative efficacy with and without vector control *Ann. Trop. Med. Parasitol.* **95**, 361-378

53. I.P. Sunish *et al.* (2007) Vector control complements mass drug administration against bancroftian filariasis in Tirukoilur, India *Bull Wld Hlth Org* **85**, 138-145

54. T.E. Erlanger, J. Keiser & J. Utzinger (2008) Effect of dengue vector control interventions on entomological parameters in developing countries: a systematic review and meta-analysis *Med. Vet. Entomol.* **22**, 203-221
